# Structural Perspectives on Extracellular Recognition and Conformational Changes of Several Type-I Transmembrane Receptors

**DOI:** 10.3389/fmolb.2020.00129

**Published:** 2020-08-07

**Authors:** Lucas M. P. Chataigner, Nadia Leloup, Bert J. C. Janssen

**Affiliations:** ^1^Crystal and Structural Chemistry, Bijvoet Center for Biomolecular Research, Faculty of Science, Utrecht University, Utrecht, Netherlands; ^2^Structural Biology and Protein Biochemistry, Morphic Therapeutic, Waltham, MA, United States

**Keywords:** structures, cell signaling, transmembrane, interactions, conformations, rearrangements

## Abstract

Type-I transmembrane proteins represent a large group of 1,412 proteins in humans with a multitude of functions in cells and tissues. They are characterized by an extracellular, or luminal, N-terminus followed by a single transmembrane helix and a cytosolic C-terminus. The domain composition and structures of the extracellular and intercellular segments differ substantially amongst its members. Most of the type-I transmembrane proteins have roles in cell signaling processes, as ligands or receptors, and in cellular adhesion. The extracellular segment often determines specificity and can control signaling and adhesion. Here we focus on recent structural understanding on how the extracellular segments of several diverse type-I transmembrane proteins engage in interactions and can undergo conformational changes for their function. Interactions at the extracellular side by proteins on the same cell or between cells are enhanced by the transmembrane setting. Extracellular conformational domain rearrangement and structural changes within domains alter the properties of the proteins and are used to regulate signaling events. The combination of structural properties and interactions can support the formation of larger-order assemblies on the membrane surface that are important for cellular adhesion and intercellular signaling.

## Introduction

Proteins at the cell surface play an important role in the formation and function of tissues. Transmembrane proteins can receive and transmit signals from the cell outside to the inside and *vice versa*, and from one cell to the other. In addition, transmembrane and membrane-associated proteins control cell-cell adhesion processes to form tissues and organs. Cell signaling and cell adhesion are dependent on protein-protein interactions at the extracellular side, protein conformations, and conformational changes play important roles in regulating these processes.

Cell-surface expressed proteins with roles in intercellular adhesion and signaling are often part of the type-I transmembrane protein group that constitutes 1,412 members in humans according to Uniprot (www.uniprot.org) (UniProt, 2019). The architecture of type-I transmembrane proteins is defined by an extracellular N-terminus, often consisting of multiple domains, followed by a single transmembrane helix, and a C-terminal intracellular segment. Other than these common features, the proteins display a great diversity in architecture, and it is this structural diversity that underlies the broad range of functionalities that has been assigned to type-I transmembrane proteins. The extracellular segment often plays a role in sensing the outside environment of a cell and in relaying communication between cells where it can act as a receiver or as a transmitter of signals. The dysfunction of cell-surface expressed type-I transmembrane proteins has been associated with a multitude of diseases ranging from developmental pathologies, immune disorders to neurological conditions and cancers. In particular the extracellular segment of these proteins is a target for drug development because of its diversity in structure, allowing specificity, and its accessibility at the outside of the cell providing access to large biologics such as antibodies (Arteaga and Engelman, 2014; Moraga et al., 2015; Large et al., 2019).

Structural biology techniques have provided detailed insights into the molecular mechanisms controlling adhesion and intercellular signaling. Structures of extracellular segments of type-I transmembrane proteins in isolation or in complexes show how these proteins interact in *cis* and in *trans* and how they can undergo conformational changes to become activated (Ferguson et al., 2003; Leloup et al., 2017, 2018; Barak et al., 2019). Most of the structural data has been obtained by X-ray diffraction studies from protein crystals, but also from NMR and cryo-electron microscopy experiments. Weak, albeit physiologically relevant, *cis* and *trans* interactions are sometimes revealed in structure determination studies that rely on crystals as these interactions can be used by the samples to form the crystal (Seiradake et al., 2010; Harrison et al., 2011; Kong et al., 2016; Pronker et al., 2016).

Here we discuss a diversity of molecular mechanisms that are used by several adhesion and intercellular signaling systems of the type-I transmembrane group of proteins in the control of adhesion and activation of signaling. We focus on the extracellular interactions and conformational changes of these type-I transmembrane proteins and discuss how structural biology techniques have been instrumental in resolving common concepts. The local environment of the proteins, at or between membranes, has an important role in the interactions and dynamics that type-I transmembrane proteins display (Jacobson et al., 2019). The combination of interactions on the same membrane, in *cis*, and between membranes, in *trans*, can drive the formation of larger-order assemblies (Seiradake et al., 2010; Harrison et al., 2011; Honig and Shapiro, 2020). The structure and interactions of the extracellular segments of type-I transmembrane proteins are controlled by pre- and post-translational modification that can drive the selectivity and affinity of the proteins in cell adhesion (Pronker et al., 2016; Chandler et al., 2019). Finally, conformational changes and rearrangement of type-I transmembrane proteins in complexes underly their control and activation as receptors in signaling processes (Ferguson et al., 2003; Kong et al., 2016; Leloup et al., 2017; Barak et al., 2019).

## Type-I Transmembrane Receptor Extracellular Interactions

### Role of Membrane Environment and Physical Constraints

To understand type-I transmembrane protein extracellular interactions, it is important to grasp the distinct molecular environment these molecules operate and evolved in. The physical forces and constraints from the membrane environment have molded topological features and architectures of type-I transmembrane proteins. Biological membranes have been described as highly complex, heterogeneous, and dynamic environments where uniquely distinct signaling and adhesion processes are mediated (Groves and Kuriyan, 2010; Honigmann and Pralle, 2016). Despite a wealth of knowledge on components such as lipids, proteins, and sugars, resolving intricacies of membrane biochemical processes has proven difficult. This is partially due to the experimental intractability of this cellular environment, and the difficulties in producing variable rich and yet well-parametrized models for *in silico* approaches. Nonetheless, a picture of the physical constraints, kinetics and thermodynamics occurring at membranes are being slowly and steadily elucidated offering insights as to the forces that shaped membrane bound proteins. In the following section we discuss relevant features of membranes that will inform discussion of type-I transmembrane protein extracellular structure and function in signaling and adhesion.

The most evident feature of biological membranes is that they provide two-dimensional (2D) fluid surfaces in which molecules can be inserted anisotropically or adsorbed reversibly (Groves and Kuriyan, 2010; Honigmann and Pralle, 2016) and this setting influences the properties of the associated molecules. The reduced entropy of transmembrane proteins supports interactions with other proteins embedded in the same membrane, because the entropic penalty for complex formation is reduced (Whitty, 2008). Such *cis* interaction may be very weak when measured in the 3D solution phase but still be relevant in the physiological 2D membrane setting (Pronker et al., 2016). From early study of signaling complexes, it was suggested that membrane as opposed to cytosolic proteins should display altered kinetics given the reduction of dimensionality of diffusible space (Wang et al., 1992; Axelrod and Wang, 1994), but also benefit from increase in probability of encounter dubbed the “local concentration effect” (Kholodenko et al., 2000). Research detailing signaling processes have since established a more nuanced picture whereby spatial temporal dynamics of membrane bound signaling molecules (Jacobson et al., 2019), and their relationship to gradients of intracellular signaling molecules, determine cellular signal interpretation (Groves and Kuriyan, 2010; Kholodenko et al., 2010). Organization of membrane proteins into functional signaling units would seem to depend on fluctuating assemblies dictated by interactions between membrane proteins, membrane lipids, soluble binding partners, and intracellular scaffolds (Kholodenko et al., 2010; Simons and Gerl, 2010).

While membranes provide a two-dimensional surface area, they are also elastic in three dimensions (3D). This deformability of membranes has been suggested to affect signaling processes. By alteration of surface to volume ratios in convex and concave protrusions, membranes may be able to control effective ligand to receptor concentrations (Schmick and Bastiaens, 2014). Membrane structure is influenced by several factors, such as intracellular scaffolding proteins and the cytoskeleton, and at the extracellular side the glycocalyx and extracellular matrix (Jacobson et al., 2019; Shurer et al., 2019). In addition, the membrane chemical composition and physical properties, such as local tension and diffusion, influence the distribution, and activity of membrane proteins (Simons and Gerl, 2010; Shi et al., 2018). Any of these properties can aid the local accumulation of transmembrane proteins. In these settings protein binding sites are likely made possible or the very least enhanced by multivalency induced by biological membranes which may regulate binding specificity and affinity (Jung et al., 2009; Csizmar et al., 2019).

At adhesion sites, where two opposing membranes are interacting through transmembrane proteins, reduced membrane thermal fluctuations (Milstein et al., 2008; Rozycki et al., 2010), and reduced intrinsic protein flexibility (Wu et al., 2011) entropically favor protein clustering. Here, a combination of proteins interacting between opposing membranes in *trans* and on the same membrane in *cis* can generate larger-order assemblies. In a crowded environment such as the membrane, for higher-order assemblies to dynamically exist in space and time requires that membrane proteins display diverse moieties to mediate specific interactions. The interactions should range in affinities to enable competitive, cooperative, and allosteric mechanisms that underlie the generation of complex signaling and adhesion patterns. Delving into what is currently known about protein interaction from high throughput approaches, it indeed appears likely that the relevant range of solution-phase affinities for interactions of membrane bound proteins (nM to mM) is broader than that of non-membrane bound proteins (Wright, 2009) and affinities can be very weak. In addition, interactions between type-I transmembrane proteins are regulated dynamically by post-translational modifications such as extracellular glycosylation which adds an additional layer of complexity to transmembrane protein complex formation.

### Weak *cis* Interactions Are Relevant for Signaling

As aforementioned, interactions of proteins found on membranes need not be of particularly high affinity when measured in the solution phase. It is notably becoming apparent through characterization of type-I transmembrane proteins that *cis* interactions can have particularly low affinities and still be relevant for signaling and adhesion events. Two striking examples in this regard are signaling and adhesion by immunoglobulin superfamily proteins KIT (Yuzawa et al., 2007) and myelin associated glycoprotein (MAG) (Pronker et al., 2016) where structural studies have paved the way to functional understanding of their respective subfamilies (receptor tyrosine kinase (RTK) class III and Siglecs). For both these proteins low affinity *cis* interactions are critical to mediate biological function.

**Figure 1:**
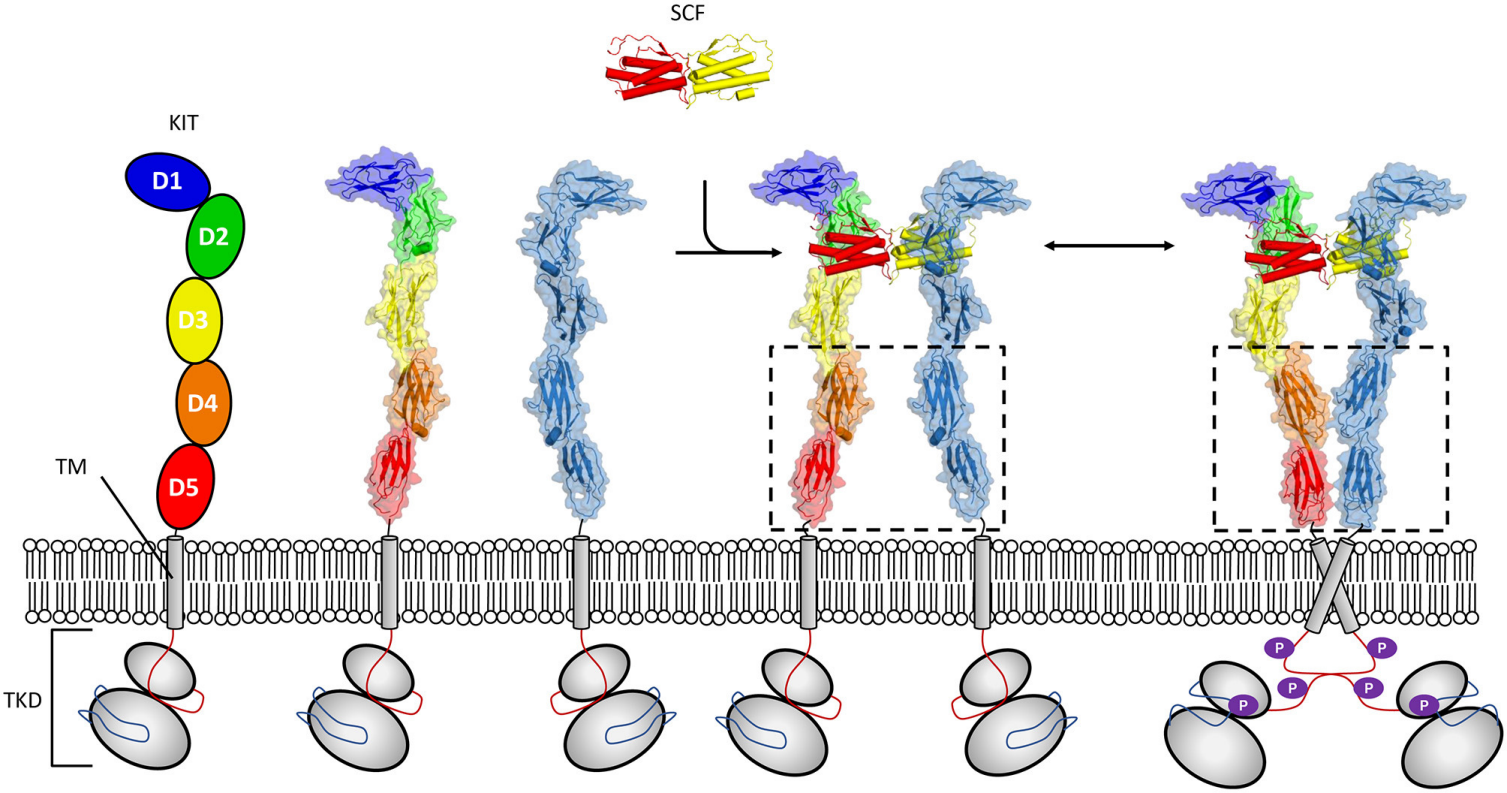
Mechanism of KIT activation by SCF. The receptor tyrosine kinases class III (RTK-IIIs) kit receptor is characterized by an ectodomain (pdb 2ec8) composed of five Ig-like domains a single membrane-spanning helix, and a conserved tyrosine kinase domain (TKD). Membrane distal domains 1–3 form a shallow groove accessible to SCF solution dimer. Binding of dimeric SCF promotes kit dimerization, facilitating conformational reorganization where weak homo *cis* interactions mediated by domains 4 and 5 contribute to establishing and/or maintaining induced kinase activity (pdb 2ew9).

Activation of KIT by cytokine stem cell factor (SCF) is critical to proper developmental time course in haematopoiesis, melanogenesis, and spermatogenesis. The study of activation of KIT by SCF illustrates well-requirements for signaling in a crowded 2D environment, since these receptors undergo ligand induced oligomerization which exploits cooperativity derived from colocalization to the membrane (Whitty, 2008) (Figure 1). Crystallographic structures have shown that dimeric SCF binds in a shallow groove formed by the membrane distal domains 1–3 of KIT. This heteromeric interaction promotes engagement of a lower affinity homomeric interaction site present at membrane proximal domains 4 and 5 in KIT (Liu et al., 2007; Yuzawa et al., 2007). The *cis* interaction occurring between KIT molecules first went unobserved in analytical ultracentrifugation sedimentation experiments of KIT extracellular segments in complex with SCF (Lemmon et al., 1997). Since, studies of full length KIT by negative stain electron microscopy (Opatowsky et al., 2014), and effects of oncogenic mutations on KIT activation have substantiated a mechanism driven by membrane derived membrane cooperativity whereby weak homotopic interactions zipper up dimer molecules into an activated state (Reshetnyak et al., 2015). Furthermore, the fingerprint motif for dimerization through salt bridges between the D4 domains of KIT as observed in crystal structures (Yuzawa et al., 2007) has been established as a common feature of RTK class III (Elegheert et al., 2011; Verstraete and Savvides, 2012; Felix et al., 2013, 2015) and V (Yang et al., 2010; Markovic-Mueller et al., 2017) receptors. Interestingly, it has been established that for other RTK III family members, homotopic contacts can be altogether stronger than those observed in KIT and form for example between unliganded colony-stimulating factor 1 receptor (CSF-1R) molecules (Elegheert et al., 2011). For other members, such as for Flt3, homotopic contacts are absent (Verstraete et al., 2011), illustrating how evolution may lead to divergent molecular interactions to regulate signaling.

The *cis* dimerization of MAG represents a second example in which a very weak solution-state interaction with a *K*_d_ of 380 μM is still relevant in a functional setting (Pronker et al., 2016). MAG is a type-I transmembrane protein expressed on the surface of cells that myelinate axons in the nervous system (Quarles, 2007). The MAG extracellular segment consists of five Ig domains and the membrane proximal Ig4 and Ig5 dimerize in an anti-parallel fashion (i.e., Ig4 interacts with Ig5 of the second molecule and *vice versa*). The dimerization of the MAG extracellular segment was first observed in the packing of crystals for structure determination and subsequently verified in solution experiments (Pronker et al., 2016; Myllykoski et al., 2018). The detailed information from the structural studies allowed to engineer the interface to generate two MAG variants, one that does not dimerize and one that dimerizes with higher affinity. Cell-based assays showed that the dimerization of MAG through domains Ig4 and Ig5 is required for signaling of MAG as a ligand into neuronal cells (Pronker et al., 2016).

### Tuning Cross-Reactive Ligand Signaling by Heteromeric Signaling Receptor Complex Formation

To provide specified output to ligand induced signaling, cell surface receptors have generally evolved highly specific molecular interactions that determine ligand selectivity (Wang et al., 2009). Yet, in complex biological systems, evolutionary pressure can also craft signaling systems with high redundancy and diversity of ligands and receptors, hallmarks of system robustness which guarantee fail safes for critical pathways (Kitano, 2004). This is well-illustrated by what is observed in innate and adaptive immunity where signaling systems display poly-specific ligands that bind both to homologous and shared receptors to form signaling complexes. A subgroup of type-I transmembrane receptors, class I cytokine receptors have been extensively characterized and mostly signal through formation of heteromeric complexes (Wang et al., 2009; Morris et al., 2018). They are responsible for cell proliferation and fate decisions of immune and hematopoietic cells. Mechanistically heteromeric complexes exploit surface-capture effects to bind various ligands with ranging affinities leading to observable redundancy and competition in signaling which in turn enable a panoply of biological responses from various target cells.

Class I cytokine receptors are composed of multiple type-I transmembrane protein chains with distinguishing conserved features and motifs. Most notably, their extracellular segments share a characteristic cytokine-binding homology region (CHR) (Bazan, 1990; Boulay et al., 2003; Verstraete et al., 2014) composed of two fibronectin type III domains (FnIII). Within the first, N-terminal, FnIII domain in the CHR region two conserved disulphide bonds are found, whereas a conserved “WSXWS” motif is found in the second FnIII domain that may have a role in folding of the protein (Bazan, 1990; Yawata et al., 1993; Boulay et al., 2003; Verstraete et al., 2014). For most receptors, cytokine binding is mediated by the joint region between FnIII domains composed of the short interdomain linker and domain interstrand loops as first outlined in the structure of the human growth hormone receptor (de Vos et al., 1992; Yawata et al., 1993; Wang et al., 2009; Morris et al., 2018). Nonetheless, various receptors also use additional domains to mediate their function. Intracellularly, these proteins have sequence motifs to allow recruitment of JAK and STAT proteins (Wang et al., 2009; Morris et al., 2018). Signaling mechanisms involving either pre-formed or ligand-induced receptor assemblies have been suggested for class I cytokine receptors (Wang et al., 2009; Kent, 2020), for heteromeric receptor assemblies the prevalent view is however that precise chain stoichiometries are dictated by specific cytokine binding. For the majority of heteromeric complexes a higher affinity ligand binding “alpha chain” is thought to recognize a specific cytokine before assembling with a “shared” chain to initiate signal transduction (Wang et al., 2009; Morris et al., 2018). Three major “shared” chains outline subgroups of heteromeric class I cytokine receptors, gp130 (Boulanger et al., 2003; Skiniotis et al., 2005), γ_c_ (Wang et al., 2005; Stauber et al., 2006), and β_c_ (Hansen et al., 2008); with other “shared” chains also used but not as prevalently (LaPorte et al., 2008; Bloch et al., 2018).

Interestingly, some of the major structural insights regarding class I cytokine receptors came from the fact that these structures form various higher-order assemblies out of ligand-bound heteromeric receptors. So while various IL4/13 (LaPorte et al., 2008) and γ_c_ (Wang et al., 2005; Stauber et al., 2006) “shared” chain assemblies form heterodimers and trimers, gp130 (Boulanger et al., 2003; Skiniotis et al., 2005), and β_c_ (Hansen et al., 2008) have additional surfaces between receptor subunits that drive ligand bound complex into forming hexameric and dodecameric assemblies relevant for signaling. Throughout, sequential assembly has been established rigorously using an array of biophysical and biochemical methods, including isothermal titration calorimetry (ITC), surface plasmon resonance (SPR), and cellular activity assays, bringing about the consensus of principles established thus far (Wang et al., 2009; Morris et al., 2018). Recent developments in the field on more unique receptors have further shone a light on even more nuanced biology of these receptors proposing allosteric regulation (Verstraete et al., 2014, 2017) and conformational selection mechanisms (Bloch et al., 2018) as driving forces for varying affinities to free and bound ligand for “shared” chains. Adding more details to our understanding of the surface-capture mechanisms of these type-I transmembrane receptors. Recent work dissecting further competition and assembly mechanisms between natural (LaPorte et al., 2008), but also synthetic cytokines (Mitra et al., 2015), or using other engineered approaches (Verstraete et al., 2017) may now pave the way to dissect cytokine signaling for therapeutic approaches and steadily bridge the gap between structural and systems-level biology of cytokine signaling.

### *Trans* Interactions: Regulating Molecular Adhesion and Recognition

Extracellular domains of membrane proteins mediating adhesion have been shaped by competing biophysical and biological constraints. On one hand, they require certain lengths, flexibilities, and affinities to satisfy conditions to establish and maintain adhesion events. While their specificities should also be finely tuneable in spatial and temporal dimensions to establish molecular recognition. Nature's remarkable solutions to this dilemma, are coming to light through breakthrough structural studies of these proteins' extracellular segments. Here, we will focus our discussion of *trans* interactions to examples drawn from the DSCAM, sidekick, and L1 family of proteins to illustrate how distinct ectodomain size, competing surfaces, and pre- and post-translational modifications influence adhesion and signaling of type-I transmembrane proteins.

**Figure 2:**
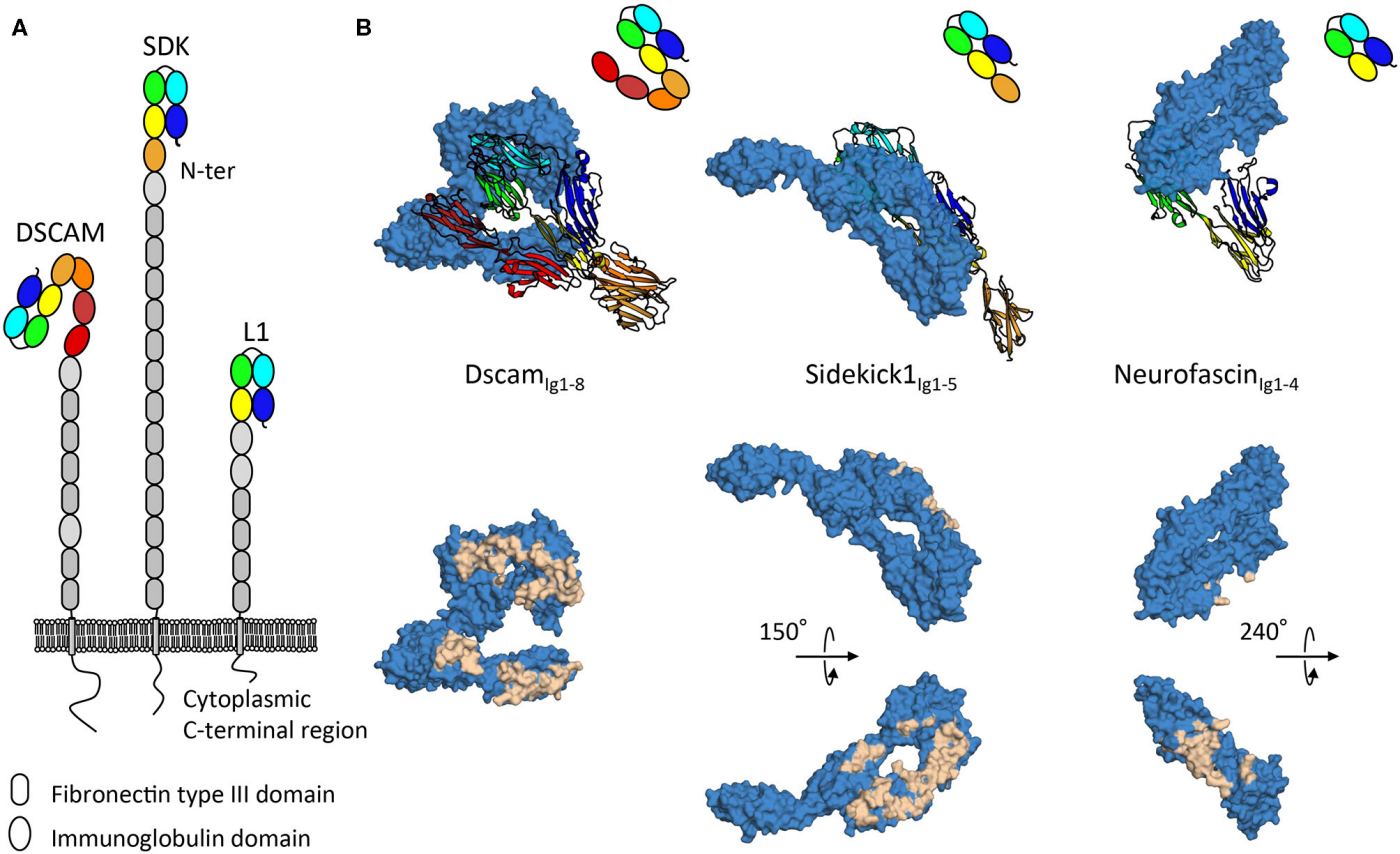
Architecture and homophilic adhesion interfaces of horseshoe containing subfamilies of the immunoglobulin superfamily. **(A)** Type-I transmembrane extracellular DSCAM, sidekick, and L1 protein families' architecture with modular domain organization comprising Ig-like and fibronectin type III repeats. **(B)** Crystal structures of homophilic binding modules of DSCAM, sidekick1, and neurofascin from DSCAM, sidekick, and L1 protein families. Top panels show how horseshoes use different faces for interactions. The ribbon copy is held in the same orientation while the space filling copy interacts with distinct faces. Bottom panels show interacting residues plotted on the surface of the space filled copy of the molecules, displaying extensive interfaces on distinct domain faces.

The DSCAM, sidekick, and L1 cell adhesion protein families are all subfamilies of the immunoglobulin superfamily and contain proteins with ectodomains composed of repeats of immunoglobulin and fibronectin type III domains (Figure 2). Intriguingly, while negative stain electron microscopy data shows the intrinsic conformational flexibility of these proteins (Schurmann et al., 2001; Meijers et al., 2007; Tang et al., 2018), as it appears that these protein families adopt elongated conformations with few domains contributing to homophilic interactions, tomographic electron microscopy data in the context of the membrane environment suggests these proteins can also neatly fit into tight adhesion interfaces (Tang et al., 2018). The specific mechanisms by which such proteins signal from distinct adhesion sites remains to date unclear. Indeed, for these various families intracellular signaling mechanisms involving interactions with scaffolding molecules (Yamagata and Sanes, 2010; Freal et al., 2019), interactions with other signaling molecules (Kiefel et al., 2011), and translocation of intracellular domains (Riedle et al., 2009; Sachse et al., 2019) have been described, nonetheless no unified downstream signaling pathway has been identified. As for how extracellular segments engage signaling, various mechanisms have been proposed. Regular patterns emerging at adhesion sites can act as a driver of signaling by protein-cluster formation (He et al., 2009). Signaling inducing constrained conformations from tight membrane apposition is another mechanism suggested on the basis of the S shaped configuration of the first eight immunoglobulin domains of DSCAM (Sawaya et al., 2008) (Figure 2). Gaining understanding of the elongated architectures of adhesion molecules and their roles in signaling remains a challenge, however progress is being made using combinations of structural techniques as discussed above.

Molecular recognition requires distinct cells be able to selectively establish interactions to transmit signals to one another (de Wit and Ghosh, 2016). From this perspective, defining specific homophilic trans interactions can be viewed as an explicit form of molecular signaling. DSCAM, sidekick, and L1 protein families remarkably all share a distinguishing architectural feature, an N-terminal horseshoe shaped binding supra-module, composed of the first four immunoglobulin domains (Figure 2) (Meijers et al., 2007; Liu et al., 2011; Goodman et al., 2016). This horseshoe feature appears to in large part define homophilic adhesion for molecules in these families, so it is noteworthy that crystallographic structures show that DSCAM, sidekick and L1 protein families use strikingly different faces of their respective horseshoe supra-module to mediate homophilic interactions (Figure 2). This would seem to indicate that, albeit likely sharing a common evolutionary origin, this module in different families has evolved very distinct extensive interaction surfaces which may be in some cases competing or complementary. It is interesting to note that homodimeric proteins have been found to have more interaction partners than non-dimers (Ispolatov et al., 2005), this seems to be well-illustrated by L1 family proteins that form promiscuous interactions with other horseshoe-containing protein families such as the contactin family (Volkmer et al., 1998).

While horseshoe containing families provide an example of the molecular diversity possible at a genomic level for molecular recognition as a form of signaling, pre-and post-translational modifications provide a further layer of complexity to the observed heterogeneity that establishes this form of signaling. Splicing is a particularly important pre-translational modification process for these molecules. For L1 family proteins such as neurofascin, over 50 distinct splice variants defined by various combinations of loop insertions, alternate domain inclusion, and linker length variations have been described that impact biological function at various developmental stages (Hassel et al., 1997; Liu et al., 2011; Kriebel et al., 2012). For Dscam, differences that regulate isoform-specific homophilic binding have been mapped to its 2nd, 3rd, and 7th immunoglobulin domains, changing homophilic binding regions on these domains offering insights into isoform-dependent binding out of thousands of possibilities (Meijers et al., 2007; Sawaya et al., 2008). Post-translational modifications of extracellular proteins, such as glycosylation, modify their surface properties, and influence their biological behavior, regulating diverse biochemical processes from protein folding to protein interactions (Moremen et al., 2012). Given inherent “stickiness” of immunoglobulin domains, glycosylation has in particular been proposed to shield unwanted interactions (Barclay, 2003). What has become more apparent for horseshoe containing proteins is that glycosylation likely plays a more constitutive role as exemplified by the patterning of L1 proteins via N-linked glycans during membrane adhesion assembly (He et al., 2009). Furthermore, they may also provide additional criteria for interaction selectivity as shown by their role in regulating neurofascin—contactin interactions (Bonnon et al., 2007). The astounding heterogeneity coming from subtle structural differences in binding interfaces of type-I transmembrane proteins mediating molecular recognition suggests that detailed structural characterization will be required to fully grasp the nuances of how they mediate their function.

## Conformational Changes Regulate Signaling

### Conformational Changes Expose Hidden Binding Sites

Proteins use conformational changes to expose or hide binding sites. In multidomain proteins such changes are often accomplished by reorganizing domains as rigid bodies with respect to each other. This inter-domain conformational change mechanism is also used by type-I transmembrane proteins to regulate their function.

**Figure 3:**
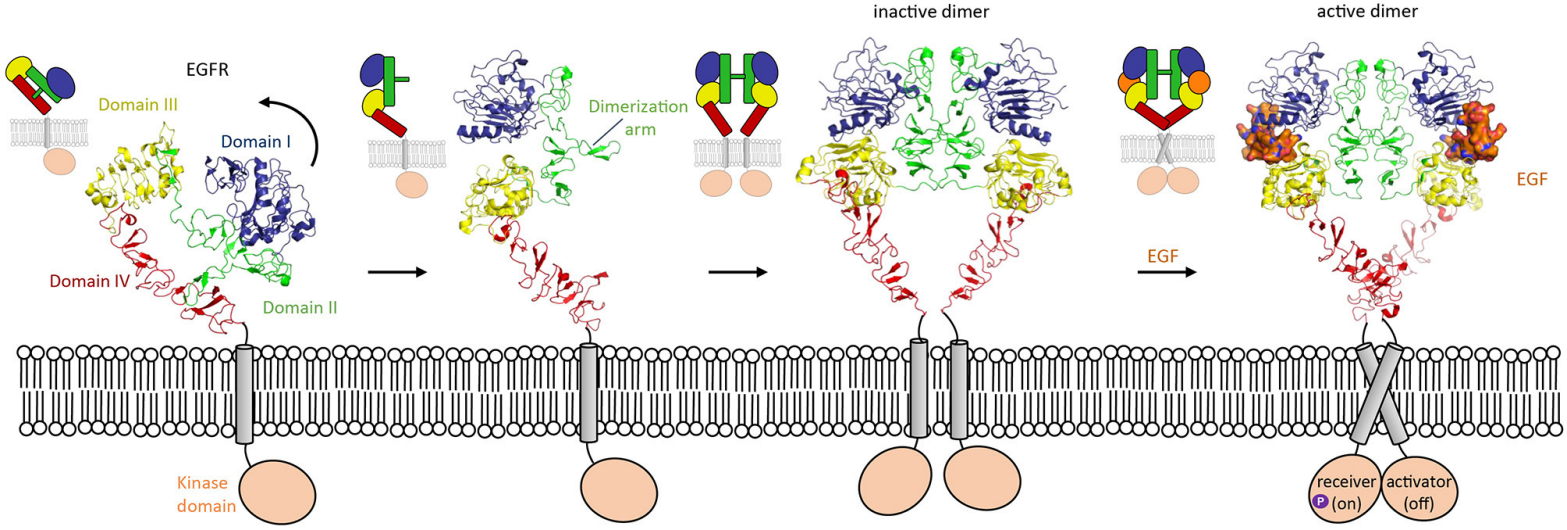
Domain rearrangement of EGFR upon activation by EGF ligand. EGFR (HER-1) undergoes a conformational change from an untethered monomer in which domain II (green) interacts with domain IV (red) (pdb 1nql), to a tethered monomer in equilibrium with an inactive homodimer (model using pdb 1n8z superposed on pdb 1ivo), and finally to an active, EGF-bound (orange) homodimer (pdb 3njp).

One of the most widely studied examples in which conformation-dependent signaling takes place is in the HER family. The HER family consists of EGFR, HER2, HER3, and HER4. HER stands for Human Epidermal growth factor Receptor, the first member of the family discovered. These receptor tyrosine kinases are involved in many processes in development, though they are most well-known for their overexpression being associated with solid tumors (Arienti et al., 2019; Khan et al., 2019; Schettini et al., 2020). The extracellular segment of these type-I transmembrane proteins consists of four domains (I, II, III, and IV). HER proteins exist on the cell surface as tethered, autoinhibited receptors. Except for HER2, for which no ligands have been identified thus far, the members of the HER family undergo a conformational change upon ligand binding. In the tethered conformation, the domain II dimerization arm interacts with domain IV (Ferguson et al., 2003). In the untethered conformation, domain II has pivoted around domain III by about 130° away from the C-terminus of the receptor, so that its dimerization arm is now exposed to the solvent (Figure 3). This untethered receptor is able to homo- or hetero-dimerize through domain II with another family member. Unliganded HER family members such as EGFR can exist on the cell membrane as inactive dimers (Chung et al., 2010; Low-Nam et al., 2011). EGFR homodimer activation is dependent on multiple factors such as interactions of the receptor with the membrane and ligand binding (Arkhipov et al., 2013). In the extended conformation, the ligand can bind in the pocket created by the proximity of domain I and III, thus stabilizing the homodimer (Lu et al., 2010) and inducing a conformational change which brings the two domains IV close by each other. These changes lead to a rearrangement of the transmembrane helixes which result in activation of the intracellular domains (Endres et al., 2013). Indeed, in absence of ligand, the intracellular module is monomeric, and self-inhibited by interactions with the membrane (Endres et al., 2013). Ligand-induced conformational changes modify the configuration of the transmembrane helixes which leads to the asymmetric dimerization of the intracellular domains (Arkhipov et al., 2013), one of which will become the activator and phosphorylate the receiver partner (Figure 3). This transphosphorylation leads to downstream signaling processes that drive cell survival and proliferation (Hubbard and Till, 2000; Lemmon and Schlessinger, 2010).

### Conformational Changes Within Domains Change Protein Surface Properties

In addition to inter-domain changes, intra-domain conformational changes can alter the properties of a protein. The recently described conformational change within the 10-bladed β-propeller of the type-I transmembrane protein sortilin, that belongs to the VPS10 family, represents a striking example of how an intradomain rearrangement can control ligand binding (Januliene et al., 2017a; Leloup et al., 2017). The pH-dependent rotation and translation of blades with respect to each other causes a reorganization of the β-propeller surface in such a way that it induces homodimerization and disrupts the interaction with several ligands.

Several members of the VPS10 family of proteins play important roles in maintaining homeostasis in our tissues. They recognize and bind ligands for internalization into a cell or for trafficking between cell compartments. These ligands also need to be released once their destination has been reached. VPS10 family members can also control neurotrophin signaling when they function by signaling as receptor together with the coreceptor p75 neurotrophic receptor (NTR) (Bothwell, 2019). The VPS10 family consists of five sorting receptors which all contain a VPS10 subunit comprising a large 10-bladed β-propeller flanked by two small stabilizing 10-CC domains. This above-average number of blades probably confers flexibility to the β-propeller, and it enables peptide ligands to bind inside its central tunnel, while larger protein partners interact with the top face of the β-propeller. Three strategies; proteolytic processing, conformational, and oligomeric changes, are employed by the receptors to prevent ill-timed binding of ligands. A common strategy for all VPS10 family members is to block the entrance of the β-propeller tunnel by their propeptide. This propeptide prevents ligand binding in the endoplasmic reticulum and early trans Golgi network (TGN) and is removed in the late TGN by the proprotein convertase furin (Munck Petersen et al., 1999).

**Figure 4:**
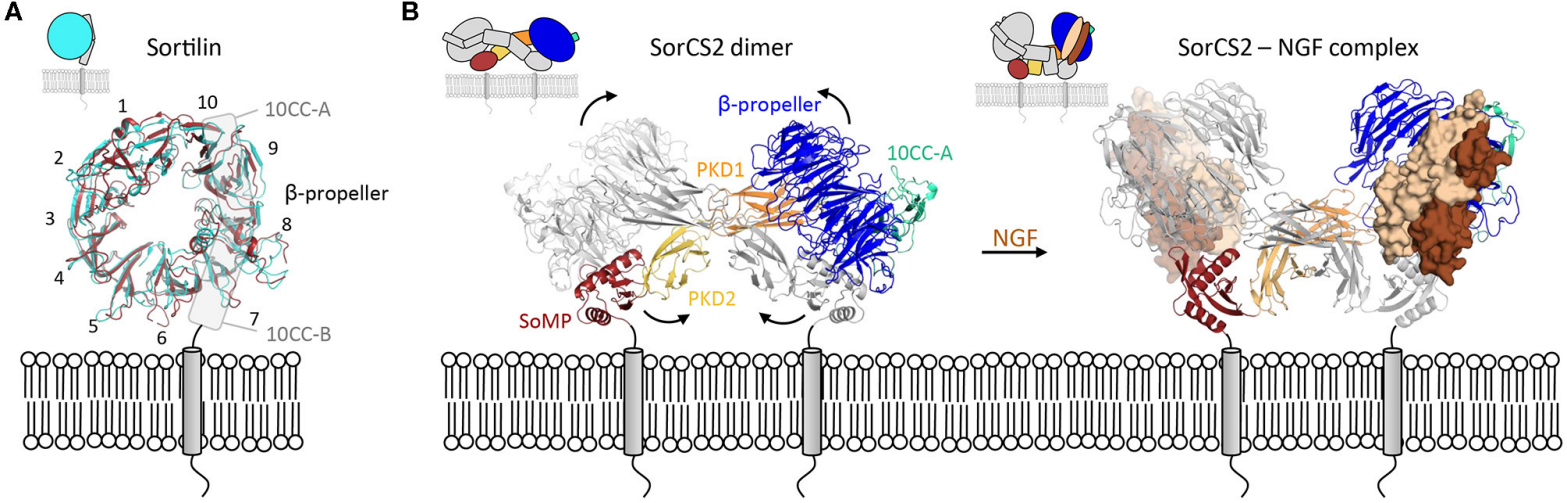
Intra- and inter-domain rearrangements underly the function of VPS10 family members. **(A)** Substantial conformational changes are apparent within the Sortilin β-propeller in changing from the monomer form (blue, pdb 3f6k) to the dimer form (red, pdb 5nmt). The 10 Sortilin β-propeller blades are numbered. **(B)** The SorCS2 dimer changes its conformation, predominantly by domain rearrangements, upon ligand binding (unbound, pdb 6ffy and bound, pdb 6fg9).

The binding of ligands to the β-propeller of Sortilin is pH-dependent. At neutral pH Sortilin is a monomer, and once its propeptide has been removed, it can bind a variety of ligands. However, as a sorting receptor, Sortilin cycles between many different cell compartments, including the TGN (pH 6.0) and early to late endosomes (pH 6.3–5.5). Upon acidification, the β-propeller of Sortilin undergoes a conformational change and its top surface, negatively charged at neutral pH, becomes more neutral (Januliene et al., 2017a; Leloup et al., 2017) (Figure 4A). These conformational and charge changes probably enable ligand release and dimerization of the receptor through its β-propeller top face, which further prevents interactions with ligands.

Except for Sortilin, members of the VPS10 family possess several domains between the VPS10 subunit and the transmembrane helix; two polycystic kidney disease (PKD) domains and a SorCS membrane proximal (SoMP) domain for the three SorCS subfamily members, an epidermal growth factor-like (EGF), 11 low-density lipoprotein receptor type A repeats (LA) and six fibronectin type III (FN3) domains for the fifth family member SorLA. One of the roles of these additional domains is to modulate binding to the VPS10 platform. For example, SorCS2 has been shown to exist in at least two different conformations (Januliene et al., 2017b; Leloup et al., 2018), in one of which ligand binding is rendered impossible by the close proximity of the ligand-binding β-propeller top face to the cell membrane (Figure 4B).

### Auto-Inhibition to Prevent Aberrant Signaling

**Figure 5:**
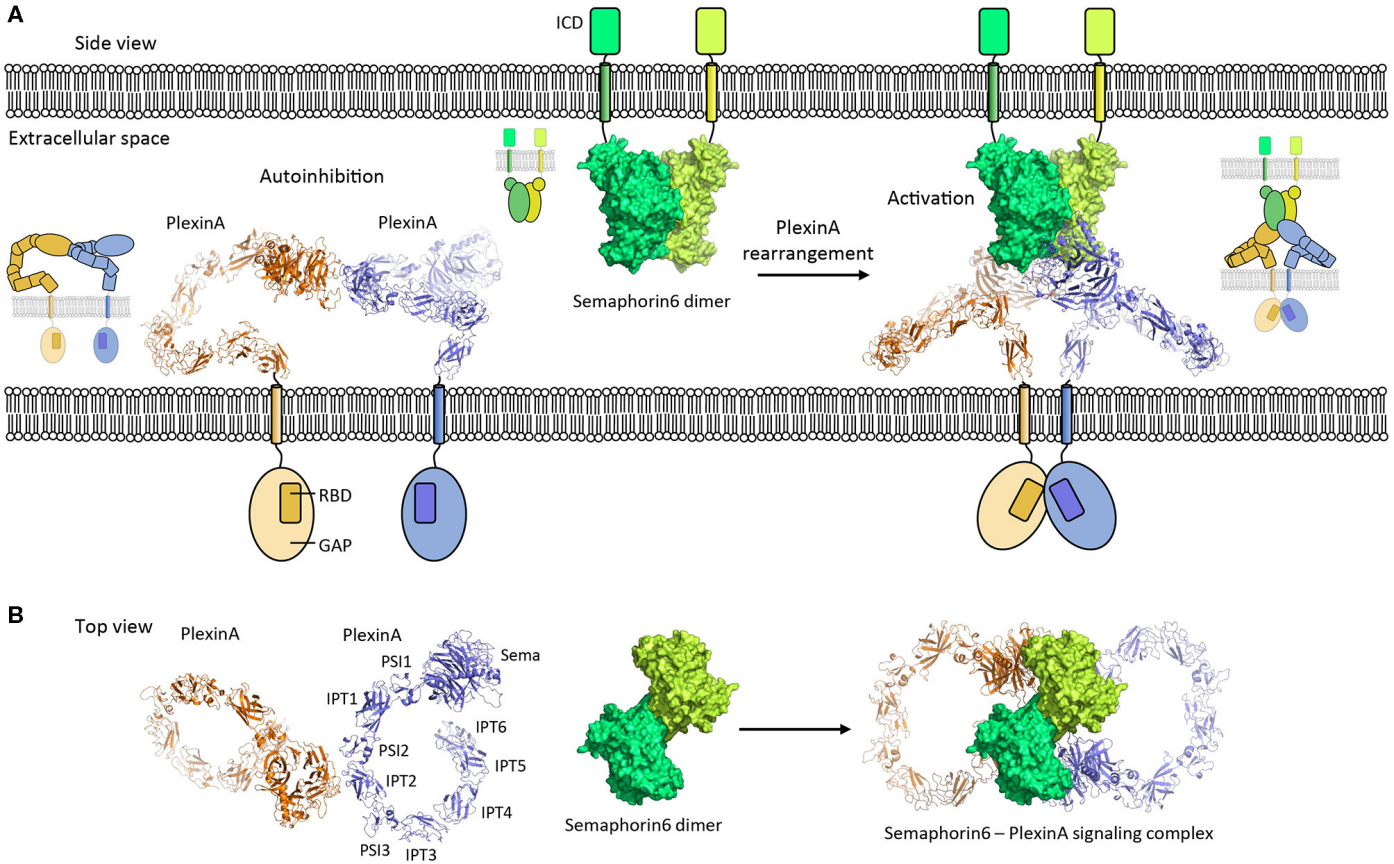
PlexinA autoinhibition model and activation by Semaphorin6 ligand. **(A)** PlexinA receptors adopt an autoinhibited state by non-symmetric *cis* dimerization (pdb 5l5k). The transmembrane helixes are separated from each other in this state. Rearrangement of the PlexinA dimer upon Semaphorin6 ligand binding (pdb 3okw) activates the PlexinA receptors by bringing the transmembrane helixes in close proximity (modeled based on 3oky and 5l5k). ICD, intracellular domain; GAP, GTPase activating protein domain; RBD, Rho GTPase binding domain. **(B)** Top view [i.e., **(A)** is rotated by 90° along the membrane]. The membranes and cytosolic segments are omitted from the panel. PSI, plexin-semaphorin-integrin; IPT, Ig domain shared by plexins and transcription factors.

**Figure 6:**
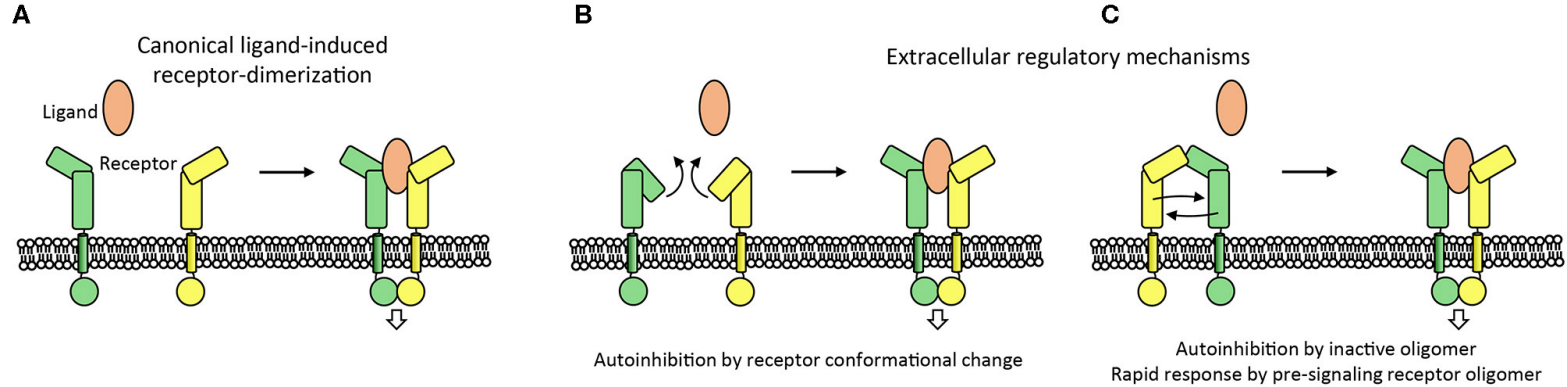
Extracellular mechanisms controlling the activation of cell signaling. **(A)** Canonical ligand-receptor system is shown in which a ligand molecule brings two receptor intracellular domains into proximity to initiate cell signaling. **(B,C)** Extracellular regulatory mechanisms that control the initiation of signaling are indicated. Receptor-conformational change or receptor-inactive oligomers can prevent aberrant initiation of signaling. In addition, the oligomerization of receptors before ligand binding may aid the receptor to respond rapidly to ligand binding by receptor reorganization **(C)**.

Ligand-induced type-I transmembrane receptor dimerization is the canonical mechanism to trigger cell signaling. Signaling in the absence of ligand is often actively prevented. This inhibition is required because receptors can encounter each other independent of ligand as a function of the local receptor concentration on the cell surface and this chance encounter may induce signaling (Atanasova and Whitty, 2012). In addition, receptors can have weak interaction sites to directly interact with each other to support ligand-induced signaling and this propensity for interaction may result in unwarranted activation. Two auto-inhibition mechanisms, in which the extracellular segment plays an important role, are commonly used by receptors to prevent ligand-independent activation; by adopting an inactive conformation and by forming inactive oligomers (Figures 5, 6). Two signaling systems that are examples for these two mechanisms; an inactive monomer conformation of HER and inactive dimers of PlexinA receptors, are discussed below.

Regulation of signaling by the conformational-change model in HER family members represents a well-described example of how an auto-inhibited conformation of a receptor prevents activation. In the auto-inhibited “compact” conformation of the HER extracellular segment, dimerization arms in domains II and IV interact and prevent high-affinity binding of the ligand (Cho and Leahy, 2002; Ferguson et al., 2003). This domain II–IV interaction stabilizes the relative orientation of domains I and III in such a way that they cannot form a high-affinity binding site for ligand binding (Figure 3). Possibly, the auto-inhibition is further strengthened by receptor dimers or larger-order clusters on the cell surface that keep the transmembrane helixes and cytosolic segments sufficiently apart to inhibit signaling (Zanetti-Domingues et al., 2018). This form of the receptor is in a dynamic equilibrium with a more extended, albeit less frequent, conformation of the receptor. The extended conformation that contains the high-affinity ligand binding site is stabilized by ligand binding. Exposure of the dimerization arms in the ligand-receptor complex enables receptor dimerization and subsequently signal triggering at the cytosolic side.

PlexinA's receptor signaling is induced by Semaphorin ligands and controls nervous system development and plasticity. The PlexinA extracellular segment has a large unusual ring-like conformation that seems to be required to bring the PlexinA cytosolic segments of two receptors into close proximity in a Semaphorin-induced receptor dimer to trigger signaling (Janssen et al., 2010; Liu et al., 2010; Nogi et al., 2010; Kong et al., 2016). Plexin dimerization induces cytosolic-segment conformational changes that activates the cytosolic Plexin GTPase activating protein (GAP) domain to enable Rap binding and subsequent Rap inactivation by catalyzing its GTP hydrolysis (Wang et al., 2012, 2013). Interestingly, the ring-like conformation is also used by the PlexinA receptor to prevent ligand independent signaling by maintaining a separation between the cytosolic PlexinA segments in a distinct head-to-stalk dimer complex of the extracellular segments (Figure 5). The autoinhibited dimer structure is rearranged upon Semaphorin ligand binding which brings the PlexinA cytosolic segments into close apposition. This activation is achieved without intramolecular conformational changes in the PlexinA extracellular segment and relies solely on rearrangement of PlexinA dimers (Figure 5).

### Poised for Signaling

The propensity of cell surface receptors to interact pre-ligand binding may serve a second role. It permits the local concentration of receptors and prepares them to respond rapidly by rearranging into a signaling competent form once ligand is bound (Figure 6C). This mechanism has, for example, been suggested for the PlexinA receptors (Kong et al., 2016), EGFR (Zanetti-Domingues et al., 2018), and for the SorCS2-p75NTR hetero-dimer receptor complex (Deinhardt et al., 2011). The auto-inhibited head-to stalk PlexinA dimer is disrupted when the Semaphorin ligand binds to one of the PlexinA receptors. The second PlexinA receptor then becomes immediately available to form the signaling competent semaphorin-plexin complex consisting of a semaphorin dimer and two PlexinA molecules. In a similar manner the EGFR receptor is pre-organized to rapidly respond to ligand although the receptors also undergo an additional intramolecular conformational change prior to signaling (Zanetti-Domingues et al., 2018). A variation on this theme is provided by the SorCS2 receptor dimer that is pre-associated with the co-receptor p75NTR. Proneurotrophin ligand binding to SorCS2 and p75NTR may separate the two receptors (Leloup et al., 2018) to trigger the dissociation of the guanine nucleotide exchange factor Trio from the cytosolic side of the SorCS2-p75NTR complex and subsequent signaling (Deinhardt et al., 2011). Whether this pre-association mechanism is essential for receptors to respond rapidly to ligand binding has, however, not been established experimentally.

It is interesting to note that whilst also homomeric class I cytokine receptors are believed to be dimers which become activated by a ligand-induced receptor conformational change (Atanasova and Whitty, 2012; Brooks et al., 2014; Waters and Brooks, 2015) and may thus be poised for signaling, it has recently been shown that, at least for three family members, the receptors exist as monomers that are dimerized by their ligands (Wilmes et al., 2020). Using carefully constructed experiments, the authors showed that the thrombopoietin receptor, the Epo receptor, and the growth hormone receptor exist as monomers at physiologically relevant cell-surface densities, and efficiently dimerize and activate upon ligand binding. Several cancer-associated mutations in these receptor systems were shown to aid ligand-independent receptor dimerization, supporting the ligand-induced receptor-dimerization activation model for these receptors (Wilmes et al., 2020). In addition, the authors determined experimentally that ligand-independent receptor dimerization is concentration-dependent and only occurs at very high cell-surface cytokine receptor densities, illustrating the importance of probing ligand-receptor activation mechanisms at physiologic concentrations. Further studies will be required to assess if and under which conditions other class I cytokine receptors are either pre-associated, or dimerized only by their ligands, and in those cases where receptor pre-association occurs whether or not this has a role in preparing the receptor system for signaling.

## Concluding Remarks

Here we have discussed several extracellular molecular mechanisms employed by type-I transmembrane proteins in cell signaling and adhesion processes (see also Figure 6). The structures and the interactions of these proteins play a critical role in the activation and control of signaling and adhesion. Structural studies have been essential in revealing the conformations, complexes, and rearrangements that underlie the function of signaling receptors and adhesion proteins. Such insights have recently provided a rationale in the design of modulators that can fine-tune signaling, [e.g., by re-orienting receptors (Moraga et al., 2015), enhancing receptor specificity and stability (Silva et al., 2019), and partially impairing receptor dimerization (Ho et al., 2017)]. These novel modulators hold promise as therapeutic candidates to treat disorders associated with aberrant receptor signaling particularly in cancers, immunotherapy, and regenerative medicine (Ho et al., 2017; Silva et al., 2019).

Several outstanding questions that center on the mechanistic principles underlying the function of type-I transmembrane proteins may be addressed in the near future by structural studies; How are the structures and interactions affected by their physiologic transmembrane setting? What is the influence of protein distribution in space and time on the organization and function of type-I transmembrane proteins? In what way are the extracellular segment and cytosolic portion of type-I transmembrane proteins coupled to organize adhesion and signaling? Currently, there is no detailed structural data that shows the direct coupling of the extracellular part of the protein with its cytosolic part, although several attempts have been made toward this endeavor (Ge et al., 2018; Uchikawa et al., 2019; Kuo et al., 2020). Most likely the transmembrane connection between the two segments, embedded in micelles or nanodisks confers too much flexibility in this setting, precluding structure solution. New techniques, such as cryo-electron tomography (Zeev-Ben-Mordehai et al., 2014) and solid-state nuclear magnetic resonance (Kaplan et al., 2016), are becoming available to study the structure and dynamics of these proteins in a transmembrane setting. Ultimately however, a combination of structural methods and cell-biology techniques is required to fully resolve the mechanistic intricacies of intercellular signaling and adhesion processes in our tissues.

## Author Contributions

All authors contributed to the conception and writing of the manuscript.

## Conflict of Interest

NL was employed by the company Morphic Therapeutic. The remaining authors declare that the research was conducted in the absence of any commercial or financial relationships that could be construed as a potential conflict of interest.
